# Supplementary material for: Women’s post-abortion contraceptive use: Are predictors the same for immediate and future uptake of contraception? Evidence from Ghana
Source: PLoS One. 2021 Dec 21;16(12):e0261005. doi: 10.1371/journal.pone.0261005 (PMC8691597; doi:10.1371/journal.pone.0261005)
Supplement: Attachment — Submitted filename: RESPONSE TO REVIEWERS.docx [file pone.0261005.s001.docx]

The Academic Editor

PLOS ONE

Dear Dr Darteh,

My co-authors and I are grateful for the opportunity to revise and resubmit our manuscript titled, “Women’s post-abortion contraceptive use: are predictors the same for immediate and future uptake of contraception? Evidence from Ghana” with manuscript number, PONE-D-20-22759.

We are writing to address the comments of the reviewers and provide your office with the revised manuscript. Please see the responses to the comments below.

Best regards,

Dr Esinam Kayi (Corresponding Author)

Reviewer #1:

*The paper was generally well written*

*The background information was well researched and unambiguous. The socio-ecological model used to underpin the study was appropriate and commendable. The objectives set were to examine key factors associated with immediate post-abortion contraceptive use and those associated with subsequent or future use. These are clear and unambiguous*

Response: We are grateful to the reviewer for these positive comments

*Secondary data was used in this study. The methods used in the primary data collection were summarised in the paper. A multi-stage sampling technique was employed. The logistic regression analysis employed was appropriate and rigorous.*

*Findings generally reflected objectives set, however, the finding that about 9% of women used contraception immediately after the abortion was wrongly reported. It is 6.9% and not 9% (see table 1).*

*The conclusions drawn and recommendations made generally reflected the objectives set and gaps respectively observed.*

Response: We are grateful for the reviewer catching this error. This has been rectified in table 1.

Reviewer #2:

*1. In the abstract, the recommendation is not specific. Again, the study did not focus on access to modern contraceptives and so the related recommendation is misplaced. Similarly, the study did not look into access to post-abortion contraceptives, hence, a wild recommendation.*

Response: The recommendation in the abstract has been rephrased to suit the findings from the study. We are grateful for this observation. Please see lines 59-62.

*2. At line 72 and 73, the statement that less than one-quarter of women in the reproductive ages (15-49) use any method of contraception is misleading: The key variable is women currently married. Again, figure is not less than one-quarter. More so, it is not ‘any method’ of contraceptive. The authors could rather include evidence of contraceptive use and induced abortion among such cohort.*

Response: The misleading information has been corrected based on evidence from the relevant references. The study consists of all reproductive aged (15-49) women (both married and unmarried) with an induced abortion in the 5 years preceding the survey. The suggestion for evidence of contraceptive use and induced abortion among the subgroup of women has been incorporated into the manuscript at lines 74-77.

*3. Data on the contraceptive 78 prevalence rate (CPR) of women who have ever had an abortion is lacking’ – this statement made on line 77 was not supported with any evidence. Apart from the GDHS (1988-2014), the GMHS (2007 and 2017) data are available.*

Response: We are grateful to the reviewer for this critical observation. The statement that the contraceptive prevalence rate (CPR) of women who have ever had an abortion is lacking has been removed. Data from the 2007 and 2017 Ghana Maternal and Health Surveys have been incorporated into the introduction sections of the manuscript. We agree that the data are available.

*4. The justification for the study is presented at line 77-88. The authors admit they did not have any nationally representative study on the subject. What about other jurisdictions – especially Africa, and other regions - Asia, Europe, Americas? Again, they reported that there are qualitative studies; they need to explore further to raise justifiable arguments.*

Response: To address this comment we have included a paragraph in the Introduction Section (line 65-72) that further argues the rationale for the study. Despite there not being any nationally representative studies on the subject, we do comment on the gaps of existing studies the reviewer suggested we mention. We have commented on studies conducted in other contexts – both quantitative and qualitative. This additional paragraph arguing the justification of our study is seen between lines 104-109.

*5. There is no commentary on the findings based on the GMHS 2007 and 2017 at the introduction section. This makes the section scanty on relevant issues.*

Response: A commentary on relevant findings based on the 2007 and 2017 GMHS have been included in the introduction section from lines 78-97.

*6. In discussing the subject with the socio-ecological model (Lines 132-160), the authors indicated three levels of interests: individual (intrapersonal), partner (interpersonal) and institutional or structural levels. The authors must therefore put the discussion under each of these interests to interpret it within the context of the model.*

Response: We appreciate this suggestion. The discussion has been placed under the individual (intrapersonal), partner (interpersonal) and institutional or structural levels in the context of the socio-ecological model at lines 308-379.

*7. At Lines 154-156, the authors indicate that the study is an explanatory study. This is questionable because there are not additional qualitative information to complement the predictor information.*

Response: We understand the reviewer’s concern about our description of the study as explanatory. We mentioned this due to the fact that our study’s aim was to examine relationships between the various factors associated with immediate and subsequent contraceptive use among abortion seekers in Ghana. By examining associations between the various independent and dependent variables we apply nomothetic explanation principles. The idiographic explanations are not possible since the data are secondary and quantitative and cannot fully get all the in-depth factors associated with immediate and subsequent use of contraception post-abortion.

*8. Information at Lines 158-160 defeat the earlier position of the author with regard to the justification of the study at Lines 77-88.*

Response: We admit that the information provided to justify the study on lines 158-160 contradicts the justification provided on lines 77-88. This error was not intended and has been removed accordingly.

*9. There are no references under the Material and Methods section; not even the source of the data sets. This is unethical or suggests plagiarism.*

Response: We regret this oversight and have included a reference of the 2017 Ghana Maternal and Health Survey report on line 182.

*10. The socio-ecological model should be discussed with the findings. This was not done at the discussion level.*

Response: We are grateful for this observation. The discussion of findings has been sectioned under the individual (intrapersonal), partner (interpersonal), and structural or institutional levels now from lines 308-379.
