# Supplementary material for: Women’s post-abortion contraceptive use: Are predictors the same for immediate and future uptake of contraception? Evidence from Ghana
Source: PLoS One. 2021 Dec 21;16(12):e0261005. doi: 10.1371/journal.pone.0261005 (PMC8691597; doi:10.1371/journal.pone.0261005)
Supplement: Attachment — Submitted filename: Re-Review of Article_Womens Post Abortion.docx [file pone.0261005.s002.docx]

**Article Type:** Research Article
**Manuscript #:** PONE-D-20-22759
**Title:** Women’s post-abortion contraceptive use: are predictors the same for immediate and future uptake of contraception? Evidence from Ghana.
**Authors:** Esinam Afi Kayi, PhD; Adriana Andrea Ewurabena Biney; Naa Dodua Dodoo; Charlotte Abra Esime Ofori; Francis Nii-Amoo Dodoo

If the article type is not Research Article, please view this page for more information on other article types: <https://journals.plos.org/plosone/s/other-article-types>

Abstract

The abstract has been revised and well written.

Introduction

Having incorporated all the suggestions, the focus of the introduction, the justification of the study and the supportive arguments are convincing.

Methods

The secondary dataset from 2017 Ghana Maternal Health Survey was used. The measures and analysis description and application have been discussed satisfactorily.

Results and discussion

The results section and the interpretations have been presented appreciable. The discussion has also been done satisfactorily using the underpinning model that guided the study.

Conclusion

Appropriately stated.
