# Supplementary material for: Women’s post-abortion contraceptive use: Are predictors the same for immediate and future uptake of contraception? Evidence from Ghana
Source: PLoS One. 2021 Dec 21;16(12):e0261005. doi: 10.1371/journal.pone.0261005 (PMC8691597; doi:10.1371/journal.pone.0261005)
Supplement: Attachment — Submitted filename: RESPONSE TO REVIEWERS.docx [file pone.0261005.s003.docx]

The Academic Editor

PLOS ONE

Dear Dr Darteh,

My co-authors and I are grateful for the opportunity to revise and resubmit our manuscript titled, “Women’s post-abortion contraceptive use: are predictors the same for immediate and future uptake of contraception? Evidence from Ghana” with manuscript number, PONE-D-20-22759R1.

We are writing to address the comments of the reviewers and provide your office with the revised manuscript. Please see the responses to the comments below.

Best regards,

Dr Esinam Kayi (Corresponding Author)

Reviewer #1:

1. *The assumption that “Given method of contraception at time of abortion as directed by a health provider” is assumed to be:*

*a. the same as actual contraceptive use*

Response: We made this assumption on the basis of the question in the dataset “did they give you the method of contraception, give you a prescription, or give you a referral?” and the corresponding options were ‘gave method’, ‘prescribed a method’, ‘gave a referral’ or ‘don’t know’. We therefore used ‘gave method’ as our measure of immediate post-abortion contraceptive use and assumed it to be actual contraceptive use and acceptance of contraception at the time of post-abortion care. Furthermore, among all the questions on post-abortion contraception, this was the only closely related and direct question asked of the post-abortion women. From these options, ‘gave method’ presupposes that the woman initiated/adopted/accepted a family planning method prior to being discharged after the abortion procedure. Given the data restrictions on the measurement of actual immediate contraceptive use after abortion, we have included this limitation in the manuscript on page 20 and modified the measures section related to immediate post-abortion contraceptive use on page 8.

*b. a measure of immediate post-abortion contraceptive use*

Response: In our study, immediate post-abortion contraceptive use was measured by the question “did they give you the method of contraception, give you a prescription, or give you a referral?” (we paraphrased the question as “given method of contraception after abortion”). According to the World Health Organization (2006, 2010), immediate use of post-abortion family planning is measured as a woman’s receipt of family planning immediately after abortion at the health facility before discharge; and or use of contraception within six months post abortion before a subsequent pregnancy. We chose to use WHO’s recommendation of immediate post-abortion contraceptive use as the dataset does not specify the timing of women’s initiation and acceptance of contraceptive use after abortion. Again, the question that most closely measures the immediacy of initiation or use of a contraceptive method is “did they give you the method of contraception, give you a prescription, or give you a referral?” We find that other studies such as Delvaux et al (2008) and McDougall et al (2009) also measure immediate post-abortion contraceptive use based on WHO’s recommendation of post-abortion contraceptive use.

2. *What post-abortion time intervals define immediate and subsequent or later contraceptive use?*

Response: The time intervals that define immediate contraceptive use after abortion is initiation or adoption of family planning immediately after abortion at the health facility before discharge; and or use of contraception within six months post abortion before a subsequent pregnancy; and subsequent or later contraceptive use after abortion is contraceptive use 6 months after abortion. For this study, From the 2017 GMHS dataset we used, the century month code for the most recent abortion was not included so we were unable to identify how many months ago women had an abortion prior to their use of contraception at the time of the survey. The information we were able to calculate was limited to the number of years ago the women had the last abortion. We found that 12.6% of women reported having an abortion within a year (or 0 years) preceding the survey. Out of this, 12.6% (or 30 women) were immediate users and 39.2% (or 93 women) were currently using a contraceptive method at the time of the survey (subsequent users). Due to this limitation of not being able to detect the actual period in months, we decided to still include these women as subsequent users of contraception as they reported current use at the time of the survey.

3. *The authors assume current contraceptive use at the time of study to be equivalent to subsequent or later contraceptive use. However, some further clarifications need to be made concerning:*

*a. How many of the 1750 immediate PAFP users continued or discontinued contraceptive use? Of those who continued use, would the authors still include them as later or subsequent PAFP users?*

Response: We were interested in the women who initiated contraception after abortion for the immediate users. From the analysis, 1750 women did not qualify to be categorized as immediate contraceptive users post abortion as only 130 women reported being given a method of contraception post-abortion. Therefore, if they continued or discontinued contraceptive use, they would be classified as subsequent PAFP users which was considered a separate event.

b. *Of those 1750 who discontinued use (if data is available), how many went on to re-use contraceptives subsequently?*

Response: From our categorization of immediate and subsequent post-abortion contraceptive users, 1750 of these women did not qualify to be classified as immediate contraceptive users following abortion. Unfortunately, the data does not allow us to analyze the proportion of women who discontinued contraceptive use in detail. From the dataset, we are only able to identify those reporting use of a method immediately and then subsequently. We found that out of the 1750 women who were not immediate users of contraception, 809 (or 46.2%) were subsequent users of post-abortion contraception.

c. *Did some of the 130 non-users of PAFP (immediate) go on subsequently to use contraceptives or not?*

Response: The 130 women were immediate PAFP users and of this number, 62.9% (82 out of 130) continued contraceptive use (subsequent users) while 37.1% (48 out of 130) were not subsequent users.

4. *In the binary logistic regression analysis, the authors did not specify whether it was a bivariate or multivariate.*

Response: In the data analysis section of the paper on page 9, we stated that we carried out multivariate analysis using two binary logistic regression models to ascertain the odds adjusting for all the factors which we deemed to be associated with immediate and subsequent post-abortion contraceptive use. However, we have modified and clarified the sentence slightly on page 14 to highlight that the results are from multivariate analyses.

5. *Further information could have been derived from a multivariate*

Response: We performed two separate multivariate regression models to examine the predictors (or factors) associated with the two primary outcome variables (immediate and subsequent post abortion contraceptive use). We believe this satisfies the information derived from the multivariate analyses.

Reviewer #2:

*After comparing the revised manuscript with the review comments, it is satisfactory to say that the authors have incorporated all the suggestions and comments into the revision. This makes the article scientifically appropriate for acceptance.*

Response: We are grateful to the reviewer for these positive comments.

*.*
